# Mapping the Evolution of Microbial-Driven Nitrogen Transformation in Inland Waters: A Bibliometric Landscape Analysis

**DOI:** 10.3390/microorganisms14040902

**Published:** 2026-04-16

**Authors:** Danhua Wang, Huijuan Feng, Hongjie Gao

**Affiliations:** 1 State Key Laboratory of Environmental Criteria and Risk Assessment, Chinese Research Academy of Environmental Sciences, Beijing 100012, China; 2 State Environmental Protection Key Laboratory of Estuarine and Coastal Environment, Chinese Research Academy of Environmental Science, Beijing 100012, China

**Keywords:** inland waters, microbial-driven, nitrogen transformation, bibliometric analysis, evolution of research hotspots, research paradigm shift

## Abstract

Inland waters are critical nodes in the global nitrogen cycle, where microbial processes govern transformations that impact water quality and ecosystem functioning. Inland waters are critical nodes in the global nitrogen cycle, where microbial processes govern transformations that impact water quality and ecosystem functioning. To systematically map the knowledge structure and to identify evolving trends in this field, a bibliometric analysis was conducted using CiteSpace on 2459 publications from the Web of Science Core Collection (1990–2024). The results reveal a significant increase in publications after 2010, peaking at 228 in 2024, with China (1541 articles) and the Chinese Academy of Sciences (776 articles) being the leading country and institution, respectively. Keyword co-occurrence and cluster analyses identify a core conceptual framework centered on microbial communities, nitrogen transformation processes (e.g., denitrification, anammox), and aquatic habitats (e.g., lakes, rivers). Based on keyword emergence and temporal trends, the analysis suggests an evolution in research focus across four dimensions: research subjects (from microbial biomass to keystone taxa), core questions (from process rates to predictive manipulation), methodological tools (from culturing to multi-omics), and mechanistic understanding (from linear pathways to complex networks). These observed patterns indicate a progressive refinement of the field. The findings provide a structured overview of the literature and may inform future research directions, but should be interpreted as bibliometric trends rather than definitive conclusions about the state of the science.

## 1. Introduction

Nitrogen is essential for aquatic ecosystems, but excessive loading from agriculture and urbanization has caused eutrophication, hypoxia, and greenhouse gas emissions in inland waters [1,2]. Microorganisms drive key nitrogen transformation processes—nitrification, denitrification, and anammox—that determine nitrogen fate [3]. Over the past three decades, molecular techniques (e.g., high-throughput sequencing, metagenomics) have generated a rapidly growing body of literature on microbial nitrogen cycling in lakes, rivers, and reservoirs [4,5].

Despite this growth, the research landscape remains fragmented. Studies are often context-dependent, methodologically diverse, and focused on specific habitats or processes, making it difficult to synthesize findings into a coherent understanding of how microbial nitrogen transformations evolve across inland waters [4,6]. This fragmentation raises two fundamental questions: What is the overall intellectual structure and historical evolution of this field? And where are the emerging frontiers? Microorganisms serve as central drivers of nitrogen cycling in inland waters, regulating the transformation and fate of nitrogen through key metabolic pathways, mainly including nitrification, denitrification, and anaerobic ammonium oxidation (anammox) [6]. These processes determine whether nitrogen is released as gaseous emissions, accumulated as pollutants, or assimilated into aquatic food webs [7]. Although early research emphasized chemical monitoring and mass-balance models to quantify nitrogen fluxes, numerous studies have now explored how microbial interactions—such as those influencing community structure, metabolic activity, and cross-species collaborations—govern nitrogen transformation efficiency. In recent years, advances in molecular biology have provided powerful tools to unravel the dark box of microbial nitrogen cycling [8]. These techniques have shifted the research focus from microbial taxonomy to functional mechanisms, uncovering adaptive nitrogen metabolic strategies in extreme environments (e.g., cold or hypersaline waters) and the effects of microbial community reassembly on nitrogen pathways in eutrophic conditions [9]. These insights have refined our understanding of microbial–nitrogen–environment interactions, establishing the field as an interdisciplinary frontier spanning environmental microbiology and biogeochemistry.

Bibliometric analysis is well-suited to address these questions. Unlike systematic reviews, which synthesize evidence on specific causal questions, bibliometrics maps large-scale publication patterns—co-authorship, keyword co-occurrence, citation bursts—to reveal hidden structures, research hotspots, and paradigm shifts over time [10]. However, bibliometrics has limitations: it cannot assess the biological validity of individual studies or provide mechanistic explanations; it reveals trends and associations, not causal mechanisms. This study therefore uses CiteSpace to analyze 2459 publications (1990–2024) on microbial-driven nitrogen transformation in inland waters. Our objectives are to: (1) map the knowledge structure and temporal evolution, (2) identify research hotspots and emerging trends, and (3) discuss observed patterns in the context of methodological and conceptual shifts. We explicitly distinguish descriptive bibliometric findings from interpretive inferences, recognizing that keyword frequencies reflect research attention, not necessarily scientific progress.

## 2. Data and Methods

### 2.1. Data Sources

This study utilized the Web of Science Core Collection (WoSCC) as the primary literature database. The search was executed on 15 December 2024, using the following exact query: TS = (“microorganism” OR “bacteria” OR “microbial community”) AND TS = (“nitrogen cycle” OR “nitrogen transformation” OR “nitrification” OR “denitrification” OR “anammox”) AND TS = (“inland water” OR “lake” OR “river” OR “reservoir”). The search was restricted to document types: Article or Review; language: English; timespan: 1990–2024. After deduplication and manual screening of irrelevant records (e.g., studies on marine systems or soil without clear inland water relevance), a final dataset of 2459 valid documents was obtained. It should also be noted that our search strategy, while capturing the mainstream literature, may have under-sampled research on certain microbial taxa (e.g., archaea, fungi) and nitrogen processes (e.g., DNRA, comammox). This limitation does not invalidate the observed shifts but calls for cautious generalization.

**Figure 1:**
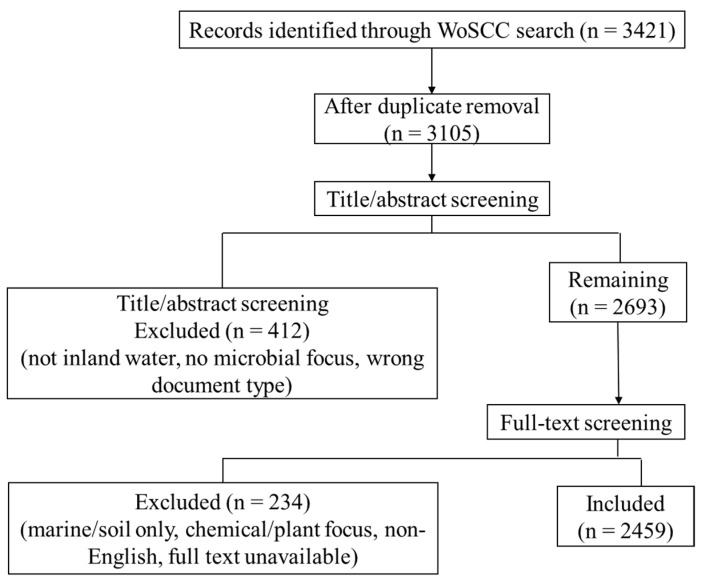
PRISMA-style flow diagram of the literature screening process.

Screening procedure: After the initial search, records were exported from WoSCC into a reference manager. Duplicates were identified using WoSCC’s “Find Duplicates” function based on title, author, journal, and publication year, followed by manual verification to ensure no unique records were erroneously removed. The remaining records were then screened in two stages. Title and abstract screening: Records were excluded if they clearly did not focus on inland waters (e.g., marine systems, soil without aquatic connection), did not involve microbial processes, or were not original research or reviews (e.g., editorials, conference abstracts, book chapters). Full-text screening: For records passing the first stage, the full text was assessed to confirm relevance to microbial-driven nitrogen transformation in inland waters. Exclusion criteria included: (i) studies exclusively on marine or terrestrial systems; (ii) studies on nitrogen transformation without microbial focus (e.g., purely chemical or plant-based); (iii) non-English articles; (iv) unavailable full text. The screening was performed semi-automatically using reference manager filters, followed by manual review by one author. A random 10% sample of the excluded records was independently checked by a second author to ensure consistency; disagreement was resolved through discussion. The final dataset comprised 2459 valid documents. A PRISMA-style flow diagram summarizing the screening process is provided in Figure 1.

### 2.2. Analytical Methods

The collected literature data from WoSCC were first analyzed using Origin 2023 to quantify annual publication trends by country and institution. Core bibliometric analysis was then performed using CiteSpace 6.1.R6 (Drexel University, Philadelphia, PA, USA) with specific parameters: annual time slicing (1990–2024, 1-year slices), node types (Author/Institution/Country/Keyword), Top 50 threshold per slice (Top N = 50), and Pathfinder network pruning combined with pruning sliced networks [11]. Keyword co-occurrence analysis was based on author keywords and Keywords Plus, supplemented by titles and abstracts for context. Singular/plural forms and spelling variants were merged using CiteSpace’s built-in alias function; a custom thesaurus file was created to normalize synonyms (e.g., “microbial community”, “bacterial community”, “microbiota” were grouped under “microbial community”). Clusters were identified using the log-likelihood ratio (LLR) algorithm. The overall network yielded a modularity Q of 0.78 (indicating a well-defined cluster structure) and a mean silhouette of 0.86 (indicating high internal consistency). For the main clusters discussed in the text, individual silhouette values ranged from 0.81 to 0.94. These indicators confirm the reliability of the clustering outputs. The analysis generated three types of outputs: (1) keyword co-occurrence networks identifying thematic associations, (2) cluster maps delineating research subfields, and (3) burst term timelines tracking emerging trends (burst detection used default parameters: γ = 1.0, minimum burst duration = 2 years). These outputs collectively enable systematic identification of research hotspots, core mechanisms, and key scientific questions in microbial-driven nitrogen transformation in inland waters. For the comparative analysis of keyword networks, the 2459 publications were divided into two groups based on the corresponding author’s country affiliation. Papers with a corresponding author affiliated in China were assigned to the “China” group; otherwise, they were assigned to the “non-China (international)” group. This classification was performed manually by inspecting author affiliation fields in WoSCC records, not algorithmically. It allows a clear comparison between research conducted by China-based teams and those from the rest of the world.

## 3. Results and Discussion

### 3.1. Spatial–Temporal Distribution and Research Pattern of Literature

#### 3.1.1. Temporal Evolution of Research Output

**Figure 2:**
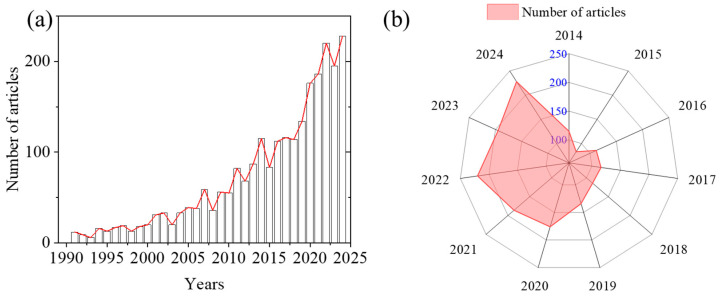
Annual number of papers published based on microbial-driven nitrogen transport and transformation in inland waters. (**a**) Number of papers published from 1990 to 2024. (**b**) The radar chart shows the trend of publications from 2014 to 2024.

As shown in the time-series data of the number of publications in this field from 1990 to 2024 (Figure 2), the evolution of its academic output exhibits distinct stage characteristics. During the initial period from 1990 to 2000, research output remained at a low level, with the average annual number of publications staying within the single digits to just over 20. This phenomenon is highly consistent with the constraints typically faced by an emerging research field in its early stages, such as a lag in theoretical system construction, insufficient research methods, and limited academic attention, indicating that the field was still in its embryonic phase of academic exploration during this time [12]. From 2000 to 2010, the number of publications showed a significant upward trend, increasing from 20 in 2000 to 55 in 2010. This reflects the gradual consolidation of the research foundation, continuous improvement in research methods and technical means, and steady growth in research output.

After 2010, the field experienced rapid growth. Publications increased from 55 in 2010 to 176 in 2020, reaching a peak of 228 in 2024. The overall trajectory shows a clear acceleration in research output during this period [13]. Between 2014 and 2024, publication numbers fluctuated, but the overall trend remained upward. The overall growth trend confirms the expanding academic influence and continuous increase in both output scale and research impact. Future output is expected to continue growing, though the rate may fluctuate due to changes in research paradigms and resource competition. It remains essential to focus not only on quantity but also on the innovation and practical application value of research. In summary, the temporal evolution of publications in this field demonstrates a clear trajectory from early exploration to accelerated development and eventual maturation. The persistent growth, especially in recent years, underscores the increasing importance of understanding microbial-driven nitrogen cycling in inland waters, reflecting its critical role in addressing eutrophication and nitrogen pollution challenges globally.

#### 3.1.2. Geographical Distribution and Institutional Contributions

**Figure 3:**
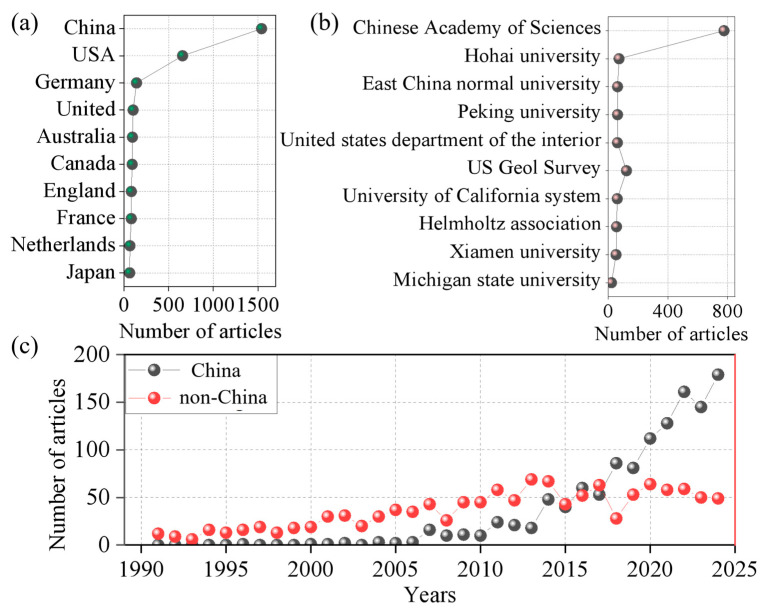
Number of papers published by different countries and institutions during 1990–2024. (**a**) A histogram of the number of articles published by different countries in this field. (**b**) A histogram of the number of articles published by different institutions in this field. (**c**) A line chart of the number of articles published in this field by China and countries other than China from 1990 to 2024.

As shown in Figure 3a, the distribution of publications across countries highlights significant spatial clustering. China holds an absolute dominant position with 1541 articles, followed by the United States with 655. Germany (139 articles), the United Kingdom (81), and France (80) also contribute significantly. Figure 3c illustrates the evolutionary trends of China and international publications from 1990 to 2024. Overall, both show increasing trends, but with clear differences in growth rhythm and scale. International publications led in the early stage (1990–2015) with stable, low growth, while Chinese output grew slowly initially but entered a phase of rapid increase after 2015. Particularly after 2020, the sharp rise in Chinese publications narrowed the gap with international totals.

These patterns may be influenced by differences in research investment, academic system maturity, and funding mechanisms across countries. However, raw publication counts have well-known limitations. First, the Web of Science Core Collection has inherent biases toward English-language journals and high-impact publications, which may underrepresent research published in other languages or regional journals. Second, multi-author international collaborations can inflate a country’s publication count if all co-authors receive credit. Third, publication volume does not directly measure research quality, innovation, or impact. Indicators such as centrality (i.e., a country’s position in collaboration networks), collaboration intensity, or normalized output (e.g., activity index) would provide a more nuanced assessment, and future bibliometric studies could incorporate these metrics.

As indicated in Figure 3b, the Chinese Academy of Sciences leads globally with 776 publications, followed by the U.S. Geological Survey with 122. Hohai University, East China Normal University, and other institutions range between 60 and 72 publications, while entities such as the University of California system and the Helmholtz Association published relatively fewer relevant papers. Currently, research in this field is highly concentrated in a few countries such as China and the United States, while many regions, particularly across developing countries and specific ecosystems, remain understudied. This imbalance may introduce spatial bias in global data collection, with certain environments being over-sampled while others with distinct conditions or acute nitrogen pollution threats are overlooked. Such geographic inequity may affect the generalizability of mechanistic insights and the accuracy of large-scale models. Enhanced international collaboration and equitable resource allocation are needed to support more representative and globally inclusive research efforts.

### 3.2. Research Hotspots Evolution and Topic Clustering

#### 3.2.1. Microbial Communities as Network Hubs in Nitrogen Cycling Research

**Figure 4:**
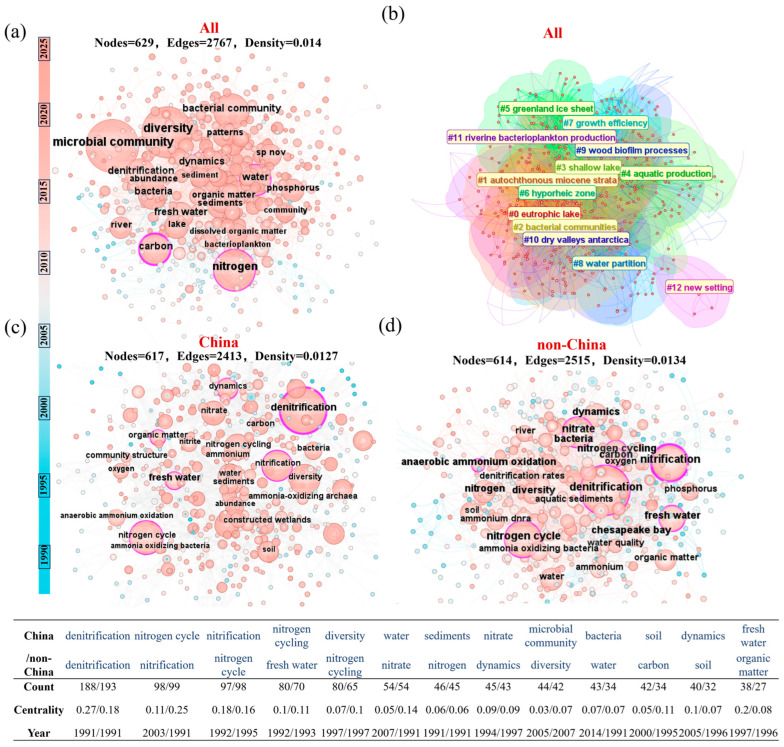
High-frequency keyword co-occurrence map and keyword clustering map of microbial-driven nitrogen transformation in inland waters. (**a**) International keyword co-occurrence map. (**b**) International keyword clustering map. (**c**,**d**) are the co-occurrence maps of China and non-China keywords, respectively.

The keyword co-occurrence network (Figure 4a) highlights microbial community and bacterial community as central nodes, emphasizing their foundational role in nitrogen cycling within inland waters. These hubs exhibit numerous connections to terms such as diversity, denitrification, and nitrification, underscoring the interdependence between community structure and nitrogen transformation efficiency [14]. Environmental terms like water, lake, and river demonstrate the influence of habitat-specific conditions (e.g., hydrology, oxygen levels) on microbial nitrogen processes. Furthermore, the co-occurrence of sediment and organic matter signifies the importance of benthic microbiomes and carbon availability in regulating nitrogen turnover, particularly through processes such as denitrification at the sediment–water interface. This network structure effectively outlines the conceptual triad of microbial community–nitrogen metabolism– aquatic habitat, anchoring microbial-driven nitrogen cycling within a coherent ecological framework [15].

#### 3.2.2. Thematic Clusters Revealing Microbial Functional Adaptations

Cluster analysis further refines our understanding of microbial nitrogen transformations across diverse environments (Figure 4b). The co-occurrence of bacterial communities (#2) and lake ecosystems (#0) in the keyword network indicates a strong research linkage between eutrophic conditions and denitrification-related keywords. This pattern could be interpreted as suggesting that researchers have frequently investigated denitrification in eutrophic lakes, potentially reflecting an ecological hypothesis that such environments favor denitrifying communities. However, bibliometric data alone cannot confirm functional selection; this remains a plausible interpretation rather than a direct finding. The presence of clusters associated with extreme environments (#5 Greenland ice sheet; #10 Antarctic dry valleys) indicates that a body of literature has investigated microbial nitrogen cycling in cold, nutrient-limited conditions. This research may contribute to understanding climate-relevant mechanisms, but the bibliometric data alone do not confirm what specific knowledge has been generated. Additional clusters such as #7 (growth efficiency) and #9 (wood biofilm processes) delve into microbial physiological and microhabitat-scale metabolic interactions, highlighting the role of microbial efficiency and biofilm-mediated processes in nitrogen assimilation and dissimilation. Together, these clusters construct a multidimensional research system that spans conventional and extreme habitats, microbial community functions, and metabolic mechanisms, providing a structured yet expanding roadmap for studying microbial-mediated nitrogen cycling [16].

#### 3.2.3. Divergent Research Emphases: China vs. Non-China

A comparative analysis of keyword networks for China-based versus non-China (international) studies reveals distinct research priorities in microbial-driven nitrogen cycling. Chinese studies (Figure 4c) display tightly connected nodes around classical metabolic pathways such as denitrification and anammox, reflecting a focused investigation into core functional microorganisms and nitrogen transformation mechanisms [17]. In contrast, the international network (Figure 4d) exhibits broader thematic linkages, integrating environmental factors like oxygen and water quality, and emphasizing multi-element coupling (e.g., carbon, phosphorus) within complex aquatic ecosystems [18]. This suggests an ecological-systemic approach that places microbial nitrogen cycling within wider biogeochemical and environmental contexts. Additionally, international research shows stronger attention to spatial heterogeneity across water types (e.g., river, freshwater), diverging from the predominantly process-oriented microbial ecology seen in Chinese studies. These complementary perspectives highlight the need to integrate deep mechanistic research—characteristic of Chinese studies—with broader ecological and multi-driver analyses to advance a holistic understanding of microbial nitrogen cycling in inland waters.

### 3.3. Research Development Context and Hot Trend

#### 3.3.1. Evolution of Research Hotspots Based on Keyword Emergence Analysis

**Figure 5:**
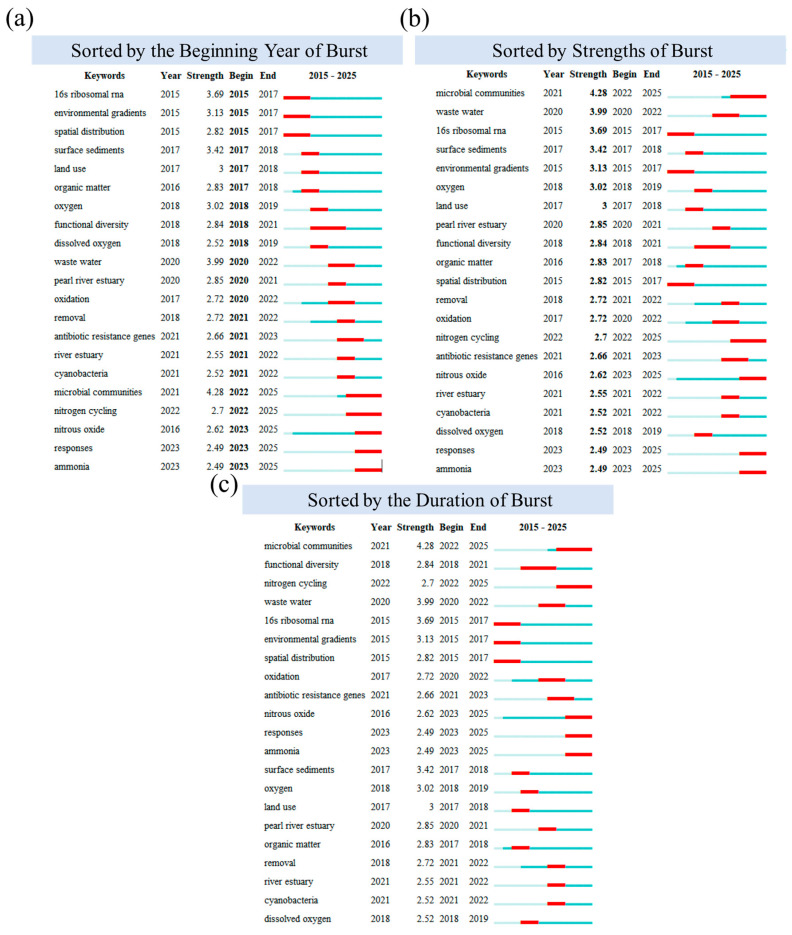
Keyword map of microbial inland water. (**a**) Sorting the burst start time of different keywords. (**b**) Sorting burst intensity of different keywords. (**c**) The burst time range intensity ranking of different keywords.

Figure 5 presents the keyword emergence map, illustrating the burst onset time, burst strength, and burst duration of key terms. From a temporal perspective, keyword bursts show a progressive sequence. Early bursts such as “nitrogen fixation” (1993–1999) reflect initial research focus on fundamental nitrogen processes and regional case studies [19]. After 2000, keywords including “dissolved organic carbon” and “ecosystem” emerged, suggesting a shift toward multi-factor coupling and ecosystem-scale investigations. In recent years (2021–2023), rising keywords such as “antibiotic resistance genes” and “microbial communities” indicate growing attention to interactions between functional microorganisms, emerging contaminants, and nitrogen cycling.

#### 3.3.2. Core and Emerging Hotspots Assessed by Burst Strength and Duration

Burst strength measures the intensity of a keyword’s sudden increase in occurrence frequency, while burst duration reflects how many consecutive years that increase persists. We used these two metrics to distinguish between “core” hotspots (high strength and long duration, indicating sustained research interest) and “emerging” hotspots (moderate strength, recent onset, and a burst continuing into the final year of the dataset, i.e., 2024). According to burst strength (Figure 5b), the strongest bursts are observed for “16s ribosomal RNA” (strength = 8.2, 2015–2020) and “microbial communities” (strength = 7.5, 2018–2024). These high-strength, sustained bursts indicate that molecular identification and community-level analysis have been central research themes. In contrast, “nitrogen cycling” (strength = 4.1, 2010–2016) and “waste water” (strength = 3.8, 2012–2018) show moderate strength and shorter duration, reflecting transient but still notable research attention [20].

Based on burst duration (Figure 5c), “nitrogen fixation” shows the longest sustained burst (1993–1999, duration = 6 years), indicating long-term foundational interest. “Dissolved organic carbon” also exhibits a prolonged burst (2005–2014, duration = 9 years), reflecting enduring attention to carbon–nitrogen coupling. For emerging hotspots, we considered keywords with a burst onset in or after 2020 that continued into 2024. These include “antibiotic resistance genes” (burst from 2021 to 2024) and “keystone taxa” (burst from 2022 to 2024). The continued presence of “microbial communities” prevailing into 2024 also suggests sustained emerging interest. It is important to note that burst detection identifies statistical anomalies in keyword frequency; it does not directly measure scientific importance or novelty. The classification into core versus emerging hotspots is a heuristic based on burst metrics and should be interpreted as a guide to research attention, not as an objective assessment of value [21].

#### 3.3.3. Thematic Evolution and Convergence Revealed Through Timeline Mapping

**Figure 6:**
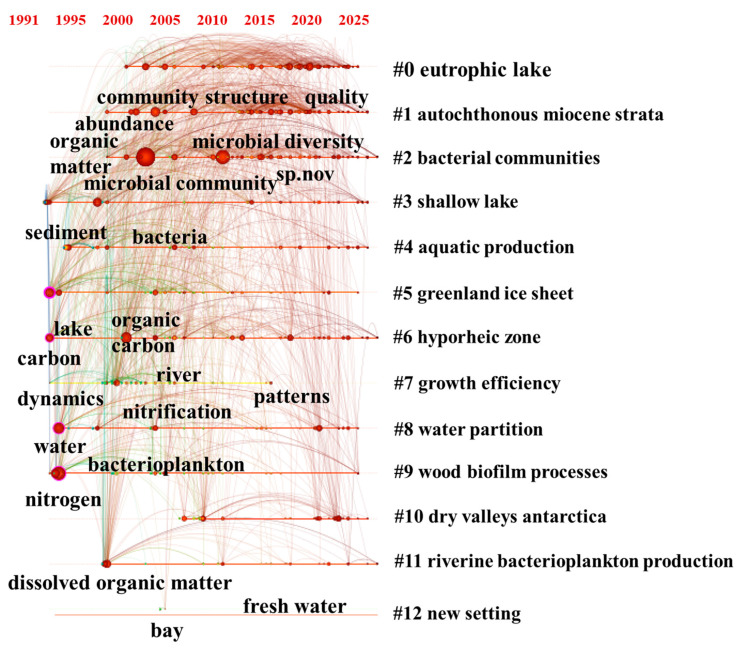
Timeline of microbial-driven nitrogen transformation in inland water.

The timeline view (Figure 6) visualizes how keyword clusters evolve over time. Each horizontal line represents a cluster (e.g., #0 eutrophic lake, #2 bacterial communities), and nodes represent keywords that appeared in that cluster during specific years. The size of a node reflects its frequency, and the position on the *x*-axis indicates the year of first significant occurrence. This visualization allows us to track the emergence, persistence, and decline of research themes [22].

From this timeline, we observe the following patterns. In the 1990s–2000s, clusters were dominated by basic terms such as “bacteria” and “nitrogen”, indicating an initial focus on taxonomic composition and nitrogen forms. During the 2000s–2010s, the term “microbial community” became central across multiple clusters, suggesting a shift from single-species to community-level analysis [23]. From 2010 onwards, keywords such as “climate change” and “functional genes” appear, indicating an expansion toward macro-environmental responses and molecular mechanisms. The persistent presence of cluster #2 (bacterial communities) across the entire timeline, with nodes progressively shifting from “diversity” (ca. 2005) to “denitrification” (ca. 2010) to “functional genes” (ca. 2018), illustrates a possible research trajectory from structural description to metabolic association to molecular regulation [23]. Similarly, cluster #0 (eutrophic lake) shows continuous association with “organic matter”, highlighting sustained interest in nitrogen–organic matter coupling under eutrophic conditions. These observations are based on keyword co-occurrence patterns and should be interpreted as trends in research attention, not as definitive claims about scientific progress.

### 3.4. The Evolution and Deepening of Hot Words in the Research Stage

As shown in Table 1, 1990–2024 is divided into four periods, revealing the core hot words of microbial-driven nitrogen transformation in inland water bodies. From 1990 to 1999, the core hot words focused on basic elements such as “biomass”, “bacterial production”, and “denitrification”. “Biomass” and “bacterial production” reflect the attention to the biomass accumulation of microbial community and the ability of bacterial growth and metabolism, which are the basic premises for analyzing microbial-driven nitrogen transformation. As a key process of nitrogen loss in inland waters, “denitrification” has become a core hot word at this stage, highlighting the focus of early research on the key migration pathways of nitrogen. The emergence of “dissolved organic carbon” and “carbon” preliminarily reflects the exploration of carbon–nitrogen coupling relationship, and constructs the initial research framework of “microorganism–nitrogen basic process–carbon–nitrogen correlation” [24]. The research is mainly oriented to reveal the basic metabolic links of the nitrogen cycle and simple biogeochemical correlation. From 2000 to 2014, the hot words expanded to “fungi”, “aquatic ecosystems”, “abundance”, “diversity” and other dimensions. The inclusion of “fungi” as a core hot word indicates that the research object has expanded from bacteria to a wider range of microbial groups such as fungi, focusing on the role of multi-bacteria in driving the nitrogen cycle; the term “aquatic ecosystems” reflects the improvement of the research scale from a single process to the ecosystem level, and the nitrogen cycle is analyzed in the framework of the structure and function of the inland water ecosystem; “abundance” and “diversity” focused on the characteristics of microbial community structure and explored the regulation of community composition and diversity on nitrogen conversion efficiency, and the hot words of environmental factors such as “heavy metals” appeared, reflecting that the research began to pay attention to the interaction between environmental factors and microbial-driven nitrogen cycle under pollution stress, showing the deepening of the research on “microbial community structure–ecosystem scale–multi-environmental factor coupling”, and gradually improving the ecological process analysis system of nitrogen cycle in inland waters.

From 2015 to 2019, molecular biology and community interaction hot words such as “16s rRNA, “gene expression” and “microbial interactions” emerged. As a classical molecular marker for microbial community analysis, “16S rRNA” has become a core hot word. With the help of molecular biotechnology, the study of “16S rRNA” can accurately analyze the composition and diversity of microbial community and break through the limitations of traditional morphology and culture methods. “Gene expression” directly points to the expression regulation of microbial functional genes, reveals the molecular mechanism of key processes of nitrogen transformation, and explains the internal logic of a microbial-driven nitrogen cycle from the genetic level. “Microbial interactions” focuses on the inter-specific collaboration and competition within the community, and analyzes the effects of microbial synergy or antagonism on nitrogen migration in complex communities. At the same time, “N_2_O production” is related to the greenhouse gas effect of the nitrogen cycle, which reflects that the research extends from process analysis to environmental effect assessment, promotes the research on the nitrogen cycle to the dimension of molecular ecology and environmental impact, and realizes the breakthrough from macro process to micro mechanism.

From 2020 to 2024, “functional prediction”, “keystone taxa” and “network analysis” became the core hot words, reflecting the trend of precise research under the deep application of omics technology. “Functional prediction” predicts the functional potential of microbial communities and predicts the occurrence trend of key processes of nitrogen cycle in advance with the help of omics data such as metagenome and meta-transcriptome data. “Keystone taxa” focuses on the key microbial groups that play a decisive role in the nitrogen cycle, identifies and analyzes their niche and metabolic contributions in the community, and realizes the precise positioning of the main body of the nitrogen cycle. By constructing microbial–environmental factors and microbial–microbial correlation networks, “network analysis” systematically analyzes the synergistic regulation of multi-factor interactions on the nitrogen cycle, and reveals the driving mechanism of the nitrogen cycle in complex systems. “Microbial processes” and “Seasonal variation” strengthen the research on the dynamic process of nitrogen cycle and the law of environmental response, while “Nutrient removal” reflects the extension of research to the application level, aiming at nitrogen pollution control and ecological restoration of inland water bodies. The frontier logic of “precise analysis of molecular mechanism–identification of key groups–multi-scale network regulation–application-oriented expansion” is presented as a whole, which promotes the evolution of nitrogen cycle research in inland waters to refinement, systematization and practicality, and provides in-depth theoretical and technical support for understanding and regulating the environmental behavior of nitrogen in inland waters.

From the stage evolution trajectory of core hot words, it can be seen that the research on microbial-driven nitrogen transformation in inland waters has gone through the development path of “basic process analysis–ecosystem coupling–molecular mechanism breakthrough–precise regulation and expansion”. In the early stage, we focused on the relationship between key nitrogen metabolism and carbon and nitrogen basis, and gradually expanded to multi-bacteria synergy, ecosystem scale and environmental factor interaction [25]. In recent years, relying on molecular technology, in-depth gene expression and community interaction has led to the integration of function prediction and network analysis to achieve precise regulation, and ultimately the application of nitrogen pollution prevention and control and ecological restoration. It has revealed a systematic deepening from macro to micro, from process to mechanism, and from foundation to application, providing a continuously expanding research framework for understanding the ecological process and environmental effects of the nitrogen cycle in inland waters.

### 3.5. The Evolution of Research Focus: Objects, Questions, and Methods

#### 3.5.1. Shifting Research Subjects: From Biomass to Keystone Taxa

The evolution of research hotspots was fundamentally driven by breakthroughs in methodological capabilities, which in turn refined the research subjects (Table 1). The early focus (1990–1999) on “biomass” and “bacterial production” relied on traditional culturing and chemical assays, treating microorganisms as a black box. The emergence of terms like “diversity” and “abundance” (2000–2014) coincided with the adoption of molecular techniques (e.g., 16S rRNA sequencing), shifting the subject to microbial community structure. The inclusion of “fungi” indicated an expansion from bacteria to broader microbial groups.

The most significant leap occurred post-2015. The rise of “microbial interactions” and “keystone taxa” (2020–2024) marks the current era, where the research subject has become the interaction network within the community and the functionally significant microorganisms that disproportionately drive nitrogen cycling. This shift suggests a strategic move toward identifying functionally significant microorganisms—sometimes referred to as “keystone taxa”—and understanding their interactions within the community. For example, recent studies using network analysis have successfully pinpointed specific bacterial taxa (e.g., certain Proteobacteria and Bacteroidetes) that disproportionately contribute to denitrification in lake sediments [26,27] illustrating how the research focus is gradually refining from bulk biomass to key players.

#### 3.5.2. Refining Core Questions: From Process Rates to Predictive Control

The core scientific questions have evolved in tandem with the shifting subjects. The early research (1990s) asked, “What are the key nitrogen transformation processes and their rates?” This is evidenced by the dominance of keywords like denitrification (Table 1), focusing on quantifying this major nitrogen loss pathway.

In the 2000–2014 period, the question expanded to, “Which environmental factors control these processes?” The appearance of keywords like heavy metals, copper, and aquatic ecosystems reflects this shift toward understanding the environmental context and constraints on microbial nitrogen cycling.

The recent period (2015–2024) has seen a growing emphasis on mechanistic and application-oriented questions, such as how microorganisms genetically and interactively achieve nitrogen transformations. While complete predictive control remains a long-term goal, there is an increasing trend toward using functional gene data to estimate process rates. For instance, metagenomic and transcriptomic approaches have been used to predict denitrification and N_2_O emission potentials in river systems [28,29], demonstrating early steps toward prediction. However, it is important to note that the appearance of keywords like “functional prediction” reflects aspiration and emerging capability rather than a fully achieved paradigm shift.

#### 3.5.3. Revolutionizing Methodologies: From Culturing to Multi-Omics and Big Data

The evolution of research hotspots was fundamentally driven by breakthroughs in methodological capabilities (See Table 1). The early focus on “biomass” and “bacterial production” (1991–1999) relied on traditional culturing and chemical assays. The emergence of terms like “diversity” and “abundance” (2000–2014) coincided with the adoption of molecular techniques (e.g., 16S rRNA sequencing). The most significant leap occurred post-2015, with the rise of “gene expression,” “functional prediction,” and “network analysis,” marking the era of high-throughput multi-omics (genomics, transcriptomics), stable isotope probing (SIP), and computational biology. These tools have greatly expanded the field’s capacity to move beyond simple taxonomic description toward functional characterization. High-throughput sequencing, metagenomics, and stable isotope probing (SIP) now allow researchers to link specific microbial taxa to nitrogen transformation activities in situ [30]. Nevertheless, challenges remain in translating functional gene abundance into actual process rates. The keyword trends indicate a clear trajectory toward multi-omics integration, but the field is still in the process of developing robust predictive frameworks.

## 4. Future Directions of Research Focus in Microbial-Driven Nitrogen Cycling

Based on the keyword burst and clustering patterns identified in our analysis, we outline several promising future research directions. Each direction is directly linked to specific bibliometric indicators and should be viewed as prospective opportunities inferred from observed trends, not as direct outputs of the analysis.

Integration of multi-omics and ecological modeling: The rising prominence of functional prediction (burst 2021–2024), gene expression (2015–2019), and network analysis (2020–2024) indicates a shift toward systems-level approaches. Future research may combine metagenomics, transcriptomics, proteomics, and metabolomics with ecological models to predict nitrogen transformation pathways and rates under changing environmental conditions. This direction is supported by the co-occurrence of microbial communities with climate change in recent timeline clusters.

Identification and engineering of keystone taxa: Keystone taxa emerged as a burst term in 2022–2024, and microbial interactions appeared in the 2020–2024 period. These trends suggest growing interest in functionally significant microorganisms. Future studies may focus on isolating, characterizing, and potentially harnessing these taxa for bioremediation. Synthetic microbial community construction and microbiome engineering are plausible next steps, although bibliometric data alone cannot confirm their feasibility.

Microbial nitrogen cycling under global change: The co-occurrence of climate change with nitrogen cycle in recent timeline clusters (2010–2024) and the persistence of seasonal variation (2020–2024) indicate increasing attention to environmental variability. Research may show an increasing focus on climate feedback mechanisms, such as N_2_O emissions under different redox conditions, and adaptive microbial responses to warming, altered precipitation, and extreme events.

Cross-ecosystem and multi-scale analyses: The presence of seasonal variation and environmental drivers (both 2020–2024) points to a need for studies spanning temporal and spatial scales. Future work could combine autonomous sensors, high-frequency sampling, and remote sensing with molecular tools to capture dynamic microbial processes at ecosystem and landscape levels.

Data science and open knowledge platforms: The burst of network analysis and functional prediction in the most recent period suggests a growing demand for computational tools and machine learning models. Openly shared datasets and collaborative platforms for data synthesis across studies and geographic regions could help address the spatial biases identified in our country-level analysis (Section 3.1.2). This direction is a forward-looking inference based on keyword trends, not a direct bibliometric finding.

In summary, the field is moving toward more predictive, application-oriented, and cross-disciplinary research. These directions should be interpreted as opportunities suggested by the evolving literature landscape, not as definitive predictions.

## 5. Conclusions

This bibliometric analysis of 2459 publications from 1990 to 2024 traces the evolution of research on microbial-driven nitrogen cycling in inland waters. The analysis robustly demonstrates four main findings. First, publication output increased slowly before 2010 and accelerated rapidly thereafter, peaking at 228 papers in 2024. Second, China with 1541 articles and the Chinese Academy of Sciences with 776 articles are the most prolific country and institution, respectively. Third, keyword co-occurrence networks center on microbial communities, nitrogen processes such as denitrification and anammox, and aquatic habitats including lakes and rivers. Fourth, burst detection reveals a temporal progression from nitrogen fixation in the 1990s to dissolved organic carbon in the 2000s to functional prediction and keystone taxa in the 2020s. Based on these patterns, we offer an interpretive synthesis of how research focus has evolved across three dimensions: research subjects moving from bulk biomass to community diversity to keystone taxa; core questions shifting from rate quantification to environmental regulation to predictive applications, though the latter remains aspirational; and methodologies advancing from culturing to molecular markers to multi-omics and network analysis. These dimensions form a heuristic framework, not a definitive paradigm shift. Several limitations constrain generalization. Our search strategy likely underrepresents research on archaea, fungi, DNRA, and comammox. Publication counts are biased toward English-language journals and Web of Science coverage. Geographic skew, with China dominating, may limit global representativeness. Bibliometric data reveal trends in research attention, not ecological processes or scientific progress per se. Future studies should adopt more comprehensive search strings, include normalized impact metrics, and integrate qualitative synthesis to validate the trends identified here. Despite its limitations, this analysis provides a structured map of the literature and may inform future research directions in microbial nitrogen transformation in inland waters.

## Figures and Tables

**Table 1:** Core hot words of microbial-driven nitrogen transformation in inland waters in different periods.

Year	Freq	Degree	Centrality	Sigma	Keyword
1991–1999	11	13	0.12	1	Biomass
	8	11	0.11	1	Bacterial production
	99	9	0.14	1	Denitrification
	28	9	0.07	1.35	Dissolved organic carbon
	92	6	0.03	1	Carbon
2000–2014	7	12	0.13	1	Fungi
	6	11	0.11	1	Aquatic ecosystems
	54	10	0.13	1	Abundance
	212	10	0.08	1	Diversity
	4	10	0.15	1	Copper
	19	9	0.07	1	Marine sediments
	12	8	0.05	1	Biodegradation
	17	8	0.05	1	Transport
	19	8	0.11	1	Heavy metals
2015–2019	4	11	0.12	1	Light
	4	9	0.09	1	Aquaculture
	4	8	0.08	1	N_2_O production
	4	8	0.08	1	Biofilms
	4	7	0.06	1	Extraction
	4	7	0.06	1	Inorganic carbon
	4	7	0.06	1	Nitrite reductase
	4	6	0.04	1	16S rRNA
	4	6	0.04	1	Deterministic processes
	4	6	0.03	1	Microbial interactions
	4	6	0.06	1	Distributions
	4	6	0.05	1	N_2_O
	4	5	0.04	1	N_2_O emissions
	4	4	0.01	1	Gene expression
2020–2024	4	9	0.07	1	Functional prediction
	4	8	0.08	1	N_2_O production
	5	3	0.01	1	Pathways
	5	2	0.01	1	Keystone taxa
	4	9	0.07	1	Functional prediction
	4	5	0.05	1	Environmental controls
	4	4	0.02	1	Nitrogen transformation
	4	2	0	1	Annotation
	6	2	0	1	Community assembly
	8	1	0	1	Ammonia
	4	5	0.03	1	Environmental drivers
	4	5	0.04	1	Methane production
	5	4	0.03	1	Microbial processes
	6	4	0.04	1	Seasonal variation
	4	2	0	1	Database
	9	1	0	1	Gradient
	8	1	0	1	Fluorescence
	8	1	0	1	Network analysis
	4	8	0.08	1	Biofilms
	4	7	0.06	1	Inorganic carbon
	4	7	0.06	1	Nitrite reductase
	4	6	0.03	1	Microbial interactions
	4	4	0.01	1	Gene expression
	4	3	0.01	1	Organic enrichment
	5	3	0.01	1	Nutrient removal

Note: The “Year” column indicates the time period, which applies to all rows below until a new year is shown. Each data row contains five values in the following order: Freq (frequency), Degree (number of co-occurrence links), Centrality (betweenness centrality, measuring a keyword’s role in connecting different clusters), Sigma (a composite index of centrality and burstness, indicating frontier potential), and Keyword. For example, “11 13 0.12 1 Biomass” reads as Freq = 11, Degree = 13, Centrality = 0.12, Sigma = 1, Keyword = Biomass.

## Data Availability

No new data were created or analyzed in this study. Data sharing is not applicable to this article.
